# Microsolvation of molecules in superfluid helium nanodroplets revealed by means of electronic spectroscopy

**DOI:** 10.3389/fchem.2014.00051

**Published:** 2014-07-16

**Authors:** Tobias Premke, Eva-Maria Wirths, Dominik Pentlehner, Ricarda Riechers, Rudolf Lehnig, Alexander Vdovin, Alkwin Slenczka

**Affiliations:** ^1^Faculty for Chemistry and Pharmacy, Institute for Physical and Theoretical Chemistry, University of RegensburgRegensburg, Germany; ^2^OSRAM Opto Semiconductors GmbHRegensburg, Germany; ^3^Carl Zeiss AGOberkochen, Germany; ^4^BASF the Chemical CompanyLudwigshafen, Germany; ^5^Philips International B.V.Amsterdam, Netherlands

**Keywords:** electronic spectroscopy, molecules, molecular complexes, microsolvation, helium droplets, zero phonon line, phonon wing

## Abstract

The empirical model explaining microsolvation of molecules in superfluid helium droplets proposes a non-superfluid helium solvation layer enclosing the dopant molecule. This model warrants an empirical explanation of any helium induced substructure resolved for electronic transitions of molecules in helium droplets. Despite a wealth of such experimental data, quantitative modeling of spectra is still in its infancy. The theoretical treatment of such many-particle systems dissolved into a quantum fluid is a challenge. Moreover, the success of theoretical activities relies also on the accuracy and self-critical communication of experimental data. This will be elucidated by a critical resume of our own experimental work done within the last ten years. We come to the conclusion that spectroscopic data and among others in particular the spectral resolution depend strongly on experimental conditions. Moreover, despite the fact that none of the helium induced fine structure speaks against the empirical model for solvation in helium droplets, in many cases an unequivocal assignment of the spectroscopic details is not possible. This ambiguity needs to be considered and a careful and critical communication of experimental results is essential in order to promote success in quantitatively understanding microsolvation in superfluid helium nanodroplets.

## 1. Introduction

One of the first helium induced fine structures reported for electronic spectroscopy in superfluid helium droplets was a doublet splitting of all zero phonon lines (ZPL) accompanied by a phonon wing (PW) with an unexpected spectral shape for tetracene (Tc) as dopant species (Hartmann et al., 1998). After the first purely empirical (Hartmann et al., 2001) and later also theoretically founded (Whitley et al., 2009) attempt to explain the doublet splitting, a new theoretical model has recently been presented, namely, coherent quantum states of the helium solvation layer covering the dopant surface (Whitley et al., 2011). With the implementation of some empirically justified modifications, this new theoretical model appeared to agree with one particular experimental spectrum (Krasnokutski et al., 2005) chosen from the wealth of experimental spectra published so far for Tc in helium droplets (Pörtner et al., 2001; Lindinger et al., 2004, 2006; Krasnokutski et al., 2005). Shortly later, a new experimental paper puts the new theoretical approach into question (Pörtner et al., 2012). There, a remarkable additional fine structure present only for the second line of the doublet of Tc provides evidence for different physical origins of the two peaks in the doublet. Moreover, the signal was found to depend on the size of the helium droplets. For very large droplets (*N* > 10^7^), the fine structure has gradually vanished and a new asymmetric peak without a fine structure grows in, however, slightly shifted to the blue. The same shift was observed for the first unstructured line in the doublet. As reported already in Pörtner et al. (2001), the full resolution of the fine structure requires a very well collimated droplet beam in combination with a single mode cw dye-laser as used in Pörtner et al. (2012). A pulsed multimode laser as used in Krasnokutski et al. (2005) does not allow for the resolution of these details. The presence of a non-superfluid helium solvation layer has already been deduced from the first rotationally resolved infrared (IR) spectrum recorded for SF_6_ in helium droplets (Hartmann et al., 1995). In contrast to vibrational or rotational excitations, electronic excitations exhibit a rather strong coupling to the helium environment. This coupling generates the PW which reveals the spectrum of elementary excitations of the helium environment. As the model of a non-superfluid helium solvation layer justifies all the helium induced fine structures recorded so far in electronic spectra, the fine structures provide evidence for the helium solvation layer. While this empirical model proposed about two decades ago is generally accepted, a quantitative simulation of the helium induced fine structures has not be seen so far. The discussion on the helium induced fine structure of Tc is the motivation for a critical presentation of our own experimental work on electronic spectroscopy of molecules in superfluid helium droplets with the focus on empirical explanations and interpretations as well as on the experimental conditions. As a result, there is no evidence speaking against the empirical model of a dopant species surrounded by a non superfluid helium solvation layer. However, the assignment for the helium induced fine structures is not as evident as presented in many papers. Moreover, experimental conditions can easily hide important details of the helium induced fine structure. This article aims to draw attention to these issues which play a key role for the quantitative understanding of microsolvation in superfluid helium droplets.

## 2. Experimental technique

The solubility for atoms and molecules in liquid helium is rather poor due to the fact that most substances condense to the solid phase at the temperature of liquid helium. This problem has been overcome by using helium droplets doped with single atoms or molecules which levitate freely in a vacuum chamber (Toennies and Vilesov, 1998). Performing chemical or physical experiments with atoms or molecules in superfluid helium droplets requires first the generation of droplets and secondly the doping of the droplets with the system to be investigated. Both conditions have successfully been investigated in the late eighties of the last century where an appropriate droplet source was combined with the well known pick-up procedure for doping of rare gas clusters (Gough et al., 1983, 1985; Lewerenz et al., 1993; Toennies and Vilesov, 1998). The droplets are generated via adiabatic expansion of helium gas under high pressure (20 bar < *p* < 100*bar*) and pre-cooled to low temperatures (4*K* < *T* < 25*K*) through a small orifice (5 μm) into a vacuum chamber (Toennies and Vilesov, 1998). Depending on the stagnation pressure and the nozzle temperature, helium droplets are generated with an average size from 10^3^ to 10^8^ helium atoms (Harms et al., 1998; Toennies and Vilesov, 2004a,b). Collimated to a droplet beam the droplets pass a skimmer to get to a second high vacuum chamber. Alternatively, a pulsed valve is used in order to generate a pulsed droplet beam. By maintaining similar gas flux the droplet density in the pulses can be significantly increased which bears advantages when using pulsed lasers. The first pulsed droplet source was a modification of a commercially available valve (General Valve No 9) (Slipchenko et al., 2002). Its performance depends critically on the nozzle shape (Yang et al., 2005; Yang and Ellis, 2008). Much higher repetition rates up to 1 kHz and more confined pulses (20 μs) are generated with a cryogenic modification of the Even Lavie valve (Even et al., 2000; Pentlehner et al., 2009). Typical expansion conditions are a stagnation pressure between 50 and 100 bar, a nozzle temperature between 10 and 30 K and an orifice of 60 μm. As in the first case the droplet beam enters the detection chamber through a skimmer with an opening diameter of 6 mm. In Regensburg two helium droplet machines are operational one with a continuous flow source and the other with a pulsed Even Lavie valve. The two machines have identical detection chambers where the droplet beam is first guided through a pick-up unit. It consists of an oven for sublimation of solid samples and of a gas cell for gas phase samples. Both have an entrance and exit aperture adjusted to the droplet beam axis. The oven is surrounded by a liquid-nitrogen cooled brass cylinder in order to shield thermal radiation and cryo-pump the effusing gas. About 10 cm downstream, the doped droplet beam is intersected perpendicularly by a laser beam. Perpendicular to both beam axes, laser induced fluorescence is collected by a lens system and imaged onto photodetectors. Two detection systems are mounted. One is a photo multiplier which records the integrated fluorescence. Second, the fluorescence is dispersed by a grating spectrograph and imaged onto the chip of a CCD (charge coupled device) camera. In the first case, the fluorescence is recorded as a function of the laser frequency which results in a fluorescence excitation spectrum. In the second case, the laser is tuned to a particular resonant absorption and a dispersed emission spectrum is recorded.

## 3. Experimental results

The signature of microsolvation is omnipresent in spectroscopy of molecules in helium droplets. In the following, our own experimental work on electronic spectroscopy of molecules or molecular aggregates inside superfluid helium nanodroplets will be reinvestigated with the focus on helium induced spectral features and their consistent interpretation. The data emerge from numerous experiments which can be separated into three groups. The first deals with the very detailed study of one particular dopant species. The second outlines comparative studies of related molecular compounds, and the final group deals with photo-chemistry inside superfluid helium droplets.

### 3.1. Electronic spectroscopy of phthalocyanine inside superfluid helium droplets

With the aim to use helium droplets as a host system to study photochemistry of cold molecules by spectroscopic means (Lehnig et al., 2009), our first experimental result drew our attention to the fundamental problem of microsolvation or, in other words, the helium induced spectroscopic features (Pentlehner et al., 2011). The corresponding dopant to helium interaction is revealed for example by a PW, a red shifted dispersed emission spectrum (Lehnig and Slenczka, 2003), or by a helium-induced fine structure as reported already for the first such spectrum (Hartmann et al., 1996b, 1998, 2001). Such spectroscopic features are also characteristic for photochemical processes. Therefore, we have studied microsolvation by means of fluorescence excitation and dispersed emission spectra first for phthalocyanine (Pc), a photochemically inactive dopant species with fortunate excitation energy, oscillator strength, and fluorescence quantum yield of the S_0_–S_1_ transition. Moreover, at that time its electronic spectroscopy was well known in the gas phase (Fitch et al., 1978, 1979, 1980, 1981), in solid matrices (Bondybey and English, 1979; Huang et al., 1982) and also in helium droplets (Hartmann, 1997). In addition to the fluorescence excitation spectrum and numerous dispersed emission spectra, our study included pump-probe spectra and the investigation of the saturation behavior. The particular experimental data revealed Pc to be surrounded by a rather rigid helium solvation layer. The entire complex moves freely inside the superfluid helium droplet. The experimental observations were as follows. The major discrepancy of the fluorescence excitation spectrum to the gas phase data was a solvation shift of the S_0_–S_1_ electrionic transition of −42 cm^−1^ (Hartmann, 1997; Hartmann et al., 2002; Lehnig et al., 2004). Otherwise, vibronic transitions appeared to be very sharp (Δν < 1 cm^−1^) with almost identical vibrational frequency as in the gas phase. The asymmetric line shape at the electronic origin with a line width in the order of 0.1 cm^−1^ reflects precisely the size distribution of the droplet beam and can be used to determine the size distribution for subcritical expansion in the continuous flow droplet source (Dick and Slenczka, 2001; Slenczka et al., 2001). For droplet sizes beyond 10^6^ helium atoms the asymmetry vanishes while the solvent shift passes a maximum and decreases with further increasing droplet size. For droplets with more than 10^7^ helium atoms a fine structure appears which can be fitted by the rotational envelop calculated for the well known almost symmetric top Hamiltonian of Pc however with increased moments of inertia as to be expected from the additional mass of the helium solvation layer (Lehnig et al., 2004; Pentlehner et al., 2009). As to be expected, the phonon wing (PW) shows a spectral structure which reveals the presence of non-superfluid helium (Hartmann et al., 2002; Lehnig and Slenczka, 2005; Lehnig et al., 2007). These details suggest that the dopant molecule is dissolved inside the droplet. Moreover, the dopant molecule is surrounded by a non-superfluid helium solvation layer.

**Figure 1:**
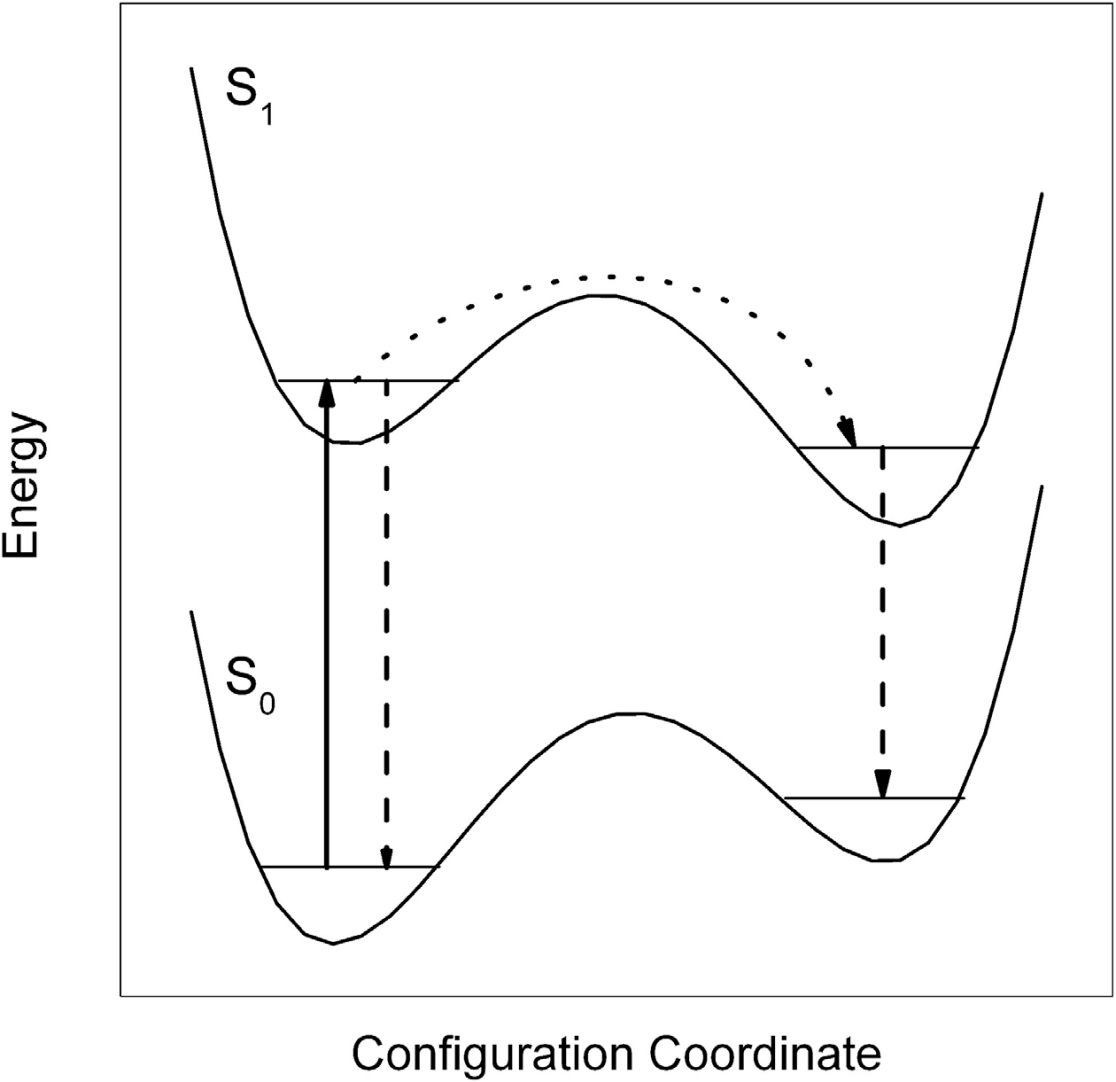
**Energetic model for the dual emission of Pc dissolved in superfluid helium droplets**. Upon electronic excitation (solid arrow) the system decays either directly (dashed arrow) or after relaxation of the helium layer configuration (dotted arrow followed by dashed arrow).

As revealed by the doubling of the entire dispersed emission spectrum, the S_0_–S_1_ electronic excitation of Pc in helium droplets transfers the excited Pc-helium complex into a metastable configuration which partly relaxes prior to radiative decay (Lehnig and Slenczka, 2003, 2004a,b) (cf. Figure 1). The corresponding branching ratio correlates with the additional excitation energy put into the vibrational degrees of freedom of the solvation complex (Lehnig and Slenczka, 2003, 2004a). Any excitation energy exceeding the electronic origin fully dissipates into the helium droplet prior to radiative decay (Lehnig and Slenczka, 2003). In the case of Pc the amount of dissipating energy promotes relaxation of the helium solvation layer. A detailed analysis of homogeneous line widths of numerous vibronic transitions did not show any correlation with the vibrational excess excitation energy. This was taken as evidence for an intermediate step preceding energy dissipation into the helium droplet, most probably internal vibrational redistribution (Pentlehner et al., 2011). The radiative decay of the relaxed complex leads to a metastable configuration in the electronic ground state (cf. Figure 1). As revealed by pump-probe experiments, the metastable configuration in S_0_ relaxes to the global minimum configuration with a rate constant of only 200 kHz (Pentlehner et al., 2011). All these findings fit to the model of a Pc-helium solvation complex which undergoes a photoinduced cycle as depicted in Figure 1. The increased moments of inertia together with the very sharp resonances in the dual emission spectra provide evidence for a helium solvation layer exhibiting a well defined configuration (which means localized helium atoms). The relaxation of the helium solvation layer which leads to the second emission spectrum is accompanied by an increase of the helium induced red shift from 42 to 52.8 cm^−1^, corresponding to a 26% increase (Lehnig and Slenczka, 2003).

Electronic excitation causes in the first place a change of the electron density distribution. In the case of Pc this change is of negligible influence on the intramolecular nuclear configuration or binding conditions. This is revealed by the close similarity of fluorescence excitation and dispersed emission spectra. However, the helium solvation layer which is soft compared to the dopant molecule may follow the change of the electron density distribution. Vice versa, the change of the electron density distribution becomes observable by helium induced spectroscopic features. The electron density distribution is an important quantity for modeling helium induced spectroscopic features. This quantity may not be properly implemented when using pair potentials as done for example in Whitley et al. (2005).

In order to learn more about the helium solvation layer we have added Ar atoms and, thus, designed Pc-Ar_*n*_ clusters inside helium droplets (Lehnig et al., 2007). Thereby, we stay with the same chromophore, namely Pc. The Ar atoms can be seen as a part of a Pc-Ar_*n*_ cluster dissolved in helium droplets or as part of the solvation layer surrounding the Pc dopant. In a sequential order a single Pc molecule and prior or afterwards a certain amount of Ar atoms were doped into the helium droplets as previously reported for Tc-Ar_*n*_ clusters (Hartmann et al., 1998). Thereby, Pc-Ar_*n*_ clusters are formed and cooled down to 0.37 K for all degrees of freedom within pico-seconds. As described in Hartmann et al. (1998) each individual sharp transition in the fluorescence excitation spectrum can be assigned to a particular cluster stoichiometry. Doping Ar atoms prior to Pc favors complexes of one Pc molecule attached to the surface of a solid Ar_*n*_-cluster while the inverse doping sequence favors complexes of one Pc molecule inside an Ar_*n*_-cluster. In the case complexes consisting of a large planar molecule (such as Pc) and only very few Ar-atoms we speak in the first case of single-sided and in the latter case of double sided Ar-occupancy. Pump-probe spectra (Hartmann et al., 1998) or dispersed emission spectra (Lehnig et al., 2007) allow to identify configurational isomers of the clusters. Using the latter technique, three complex configurations were identified for the Pc-Ar cluster exhibiting Ar-induced red shifts of 15, 4, and 1.6 cm^−1^, respectively. The vibrational fine structure of the most abundant cluster was identical to bare Pc in helium droplets and its Ar-induced red shift of 15 cm^−1^ was identical as reported for the corresponding gas phase experiment (Cho and Kim, 2000). This speaks for a complex configuration with an Ar atom just above the center of the π-conjugated ring close to the center of mass of Pc, a position coincident with the global minimum of the Pc-Ar pair potential which amounts to roughly 680 cm^−1^ in binding energy (Cho and Kim, 2000; Lehnig et al., 2007). Upon vibronic excitation with excess energy of only 128 cm^−1^ put into a low energy vibrational mode of this Pc-Ar cluster (which is less than 20% of the dissociation energy of the isolated Pc-Ar cluster), emission of bare Pc could be recorded in addition to the cluster emission (Lehnig et al., 2007). Further dynamics upon electronic excitation has been observed for the Pc-Ar_2_ clusters. It is the smallest cluster which allows for distinguishing single-sided and double-sided Ar-occupancy on the planar Pc dopant: the former favored by the doping sequence of Pc-Ar, while the latter favored by the doping sequence of Ar-Pc. For one of the most prominent signals of a single-sided Pc-Ar_2_ cluster, the dispersed emission spectrum recorded upon excitation at vibronic transitions showed dual emission. In addition to the ordinary emission spectrum identical to that upon excitation at the corresponding electronic origin, a second emission spectrum was observed matching in the frequency and intensity distribution perfectly with the dispersed emission upon excitation at the origin of a double-sided Pc-Ar_2_ cluster (Lehnig et al., 2007).

At this point one may raise the question on the structure of the solvated clusters. Are we dealing with Pc-Ar_*n*_ complexes surrounded by a helium solvation layer or may there be Ar atoms attached to the helium solvation layer of Pc? In the first case Ar atoms are merged into the helium solvation layer while in the second case the Pc-helium complex remains intact and the Ar atom is separated from the dopant by the helium solvation layer. It is not only the small red shift of only 1.6 and 4 cm^−1^ not reported for the gas phase experiment which provides evidence for the latter complex. It is also the emission of bare Pc recorded upon excitation of a Pc-Ar cluster with an excess excitation energy of only 128 cm^−1^ (cf. previous paragraph) and the configurational modification from a single-sided to a double-sided Pc-Ar_2_ complex induced by electronic excitation which reveals a rather small binding energy as to be expected for Pc and Ar shielded from each other by the helium layer. It should be noted that Pc-Ar clusters in helium droplets exhibit a similar relaxation dynamics upon electronic excitation as depicted in Figure 1 for bare Pc in helium droplets (Lehnig and Slenczka, 2004a). As the change of the electron density distribution accomplishes the relaxation of the helium solvation layer it may also afford the dissociation of the van der Waals clusters inside helium droplets.

### 3.2. Comparative studies of related molecular compounds

While electronic spectra of Phthalocyanines show very sharp transitions, other dopant species have shown surprisingly severe line broadening in the electronic spectra recorded in helium droplets. This may be due to damping of vibrational excitations in particular of low energy and large amplitude modes or due to perturbation of the change of the electron density distribution. Much information on helium induced line broadening was provided by systematic investigations of a series of related dopant species. For three molecular species namely Pyrromethene (Pentlehner et al., 2011; Stromeck-Faderl et al., 2011), Porphyrin (Pentlehner et al., 2011; Riechers et al., 2013), and Anthracene (Pentlehner et al., 2010, 2011; Pentlehner and Slenczka, 2012, 2013) several derivatives have been investigated which differ in the number and the species of substituents such as methyl, ethyl, propyl, phenyl, and cyano groups which substitute hydrogen atoms in the periphery of the molecular compound. The main conclusions concerning the influence of electronic and vibrational degrees of freedom will be outlined for each of the three molecular species.

**Figure 2:**
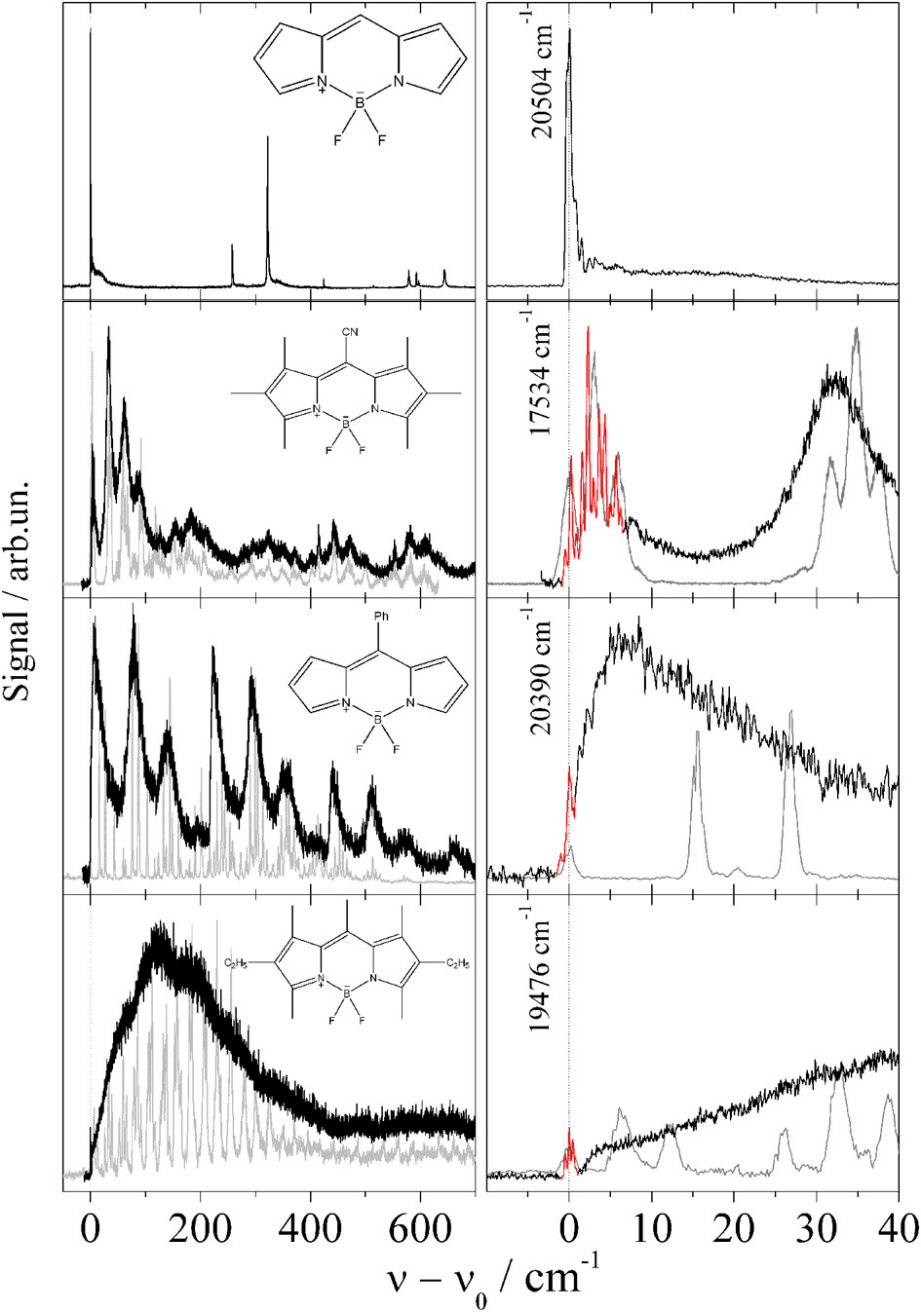
**Fluorescence excitation spectrum of four Pyrromethene drivatives in helium droplets (black and red)**. For comparison the supersonic jet spectra are added in gray corrected for the helium solvation shift. Extended low energy progressions are damped in helium droplets (**left panel**) while the electronic origin is not (**right panel**).

The series of Pyrromethene dye molecules includes derivatives such as 1,2,3,5,6,7-hexamethyl-8-cyanopyrromethene-difluoroborat, 8-phenylpyrromethene-difluoroborat, and 1,3,5,7,8-pentamethyl-2,6-diethyl-pyrromethen-difluoroborat. If one disregards intramolecular configurational variants of the substituted derivatives, the symmetry of the Pyrromethene derivatives listed in Figure 2 is identical to the non-substituted compound shown in the top panel. For all derivatives the substitution is accompanied by extended progressions of torsional and/or bending modes which are well resolved in the gas phase (Stromeck-Faderl et al., 2011) (cf. Figure 2 left panel gray lines). Extended progressions reveal different equilibrium configuration of the substituents in the two electronic states. When put into helium droplets, the corresponding progressions look like the gas phase spectrum convoluted with a line broadening function (cf. Figure 2 left panel black lines) (Pentlehner et al., 2011). It should be noted that in Figure 2 the helium induced solvent shift of the electronic spectra has been ignored in order to compare the vibrational fine structure of both spectra. In contrast to the torsional mode progressions, the electronic origin remains spectrally sharp (cf. Figure 2 right panel red line). In some cases (second and bottom panel in Figure 2) a fine structure is recorded which could not be resolved in the gas phase. These observations provide clear evidence for line broadening due to damping of vibrational modes by the helium environment, a mechanism which leaves the electronic origin unaffected. Thus, in the case of the Pyrromethene derivatives the vibrational degrees of freedom and in particular those of the substituents suffer from helium induced line broadening while purely electronic excitations do not (Pentlehner et al., 2011; Stromeck-Faderl et al., 2011).

**Figure 3:**
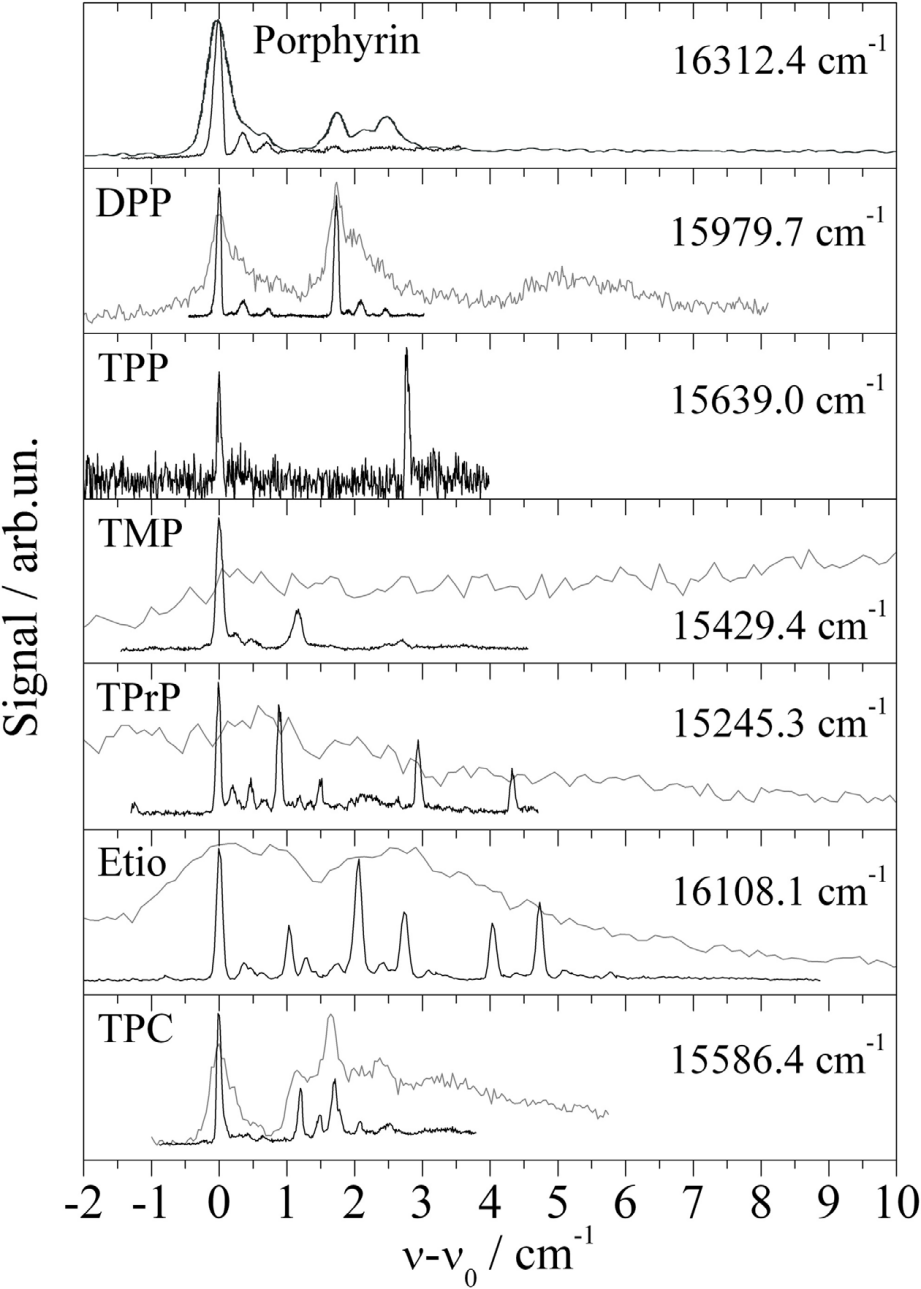
**Electronic origin of six Porphyrin derivatives, Diphenylporphyrin (DPP), Tetraphenylporphyrin (TPP), Tetramethylporphyrin (TMP), Tetrapropylporphyrin (TPrP), Tetraethyltetramethylporphyrin (Etio), and one chlorine compound Tetraphenylchlorin (TPC)**. For high photon flux (gray lines) saturation broadening and intense PW hides the fine structure (black line) observed for greatly reduced photon flux.

The study of Porphyrin (Riechers et al., 2013) includes derivatives such as 5,15-diphenylporphyrin (DPP), 5,10,15,20-tetraphenylporphyrin (TPP), 5,10,15,20-tetramethylporphyrin (TMP), 5,10,15,20-tetrapropylporphyrin (TPrP), and 2,7,12,27-tetraethyl-3,8,13,18-tetramethylporphyrin (Etio). In addition 5,10,15,20-tetraphenylchlorine (TPC) was investigated, which came as an impurity of the TPP sample (Riechers et al., 2013). Again, for all derivatives the molecular symmetry is conserved if one ignores the configurational variants of the substituents. None of the Porphyrin derivatives shows signals which could be attributed to an envelope or fully resolved progression of low energy modes representing torsional or bending modes of the substituents. Obviously, the equilibrium configuration of the substitunets is maintained upon electronic excitation as is the nuclear configuration of the Porphyrin moiety (Riechers et al., 2013). In contrast to the spectra recorded by means of a pulsed dye laser (Lindinger et al., 2001; Lehnig et al., 2007), the low photon flux and single mode radiation of a cw-dye laser enables resolution of a triple peaked ZPL of Porphyrin as shown in the top panel of Figure 3. The series of Porphyrin derivatives including the TPC compound exhibit this triple peak feature with slight modifications for each Porphyrin derivative (for more details cf. Riechers et al., 2013). For DPP the triple peak feature doubles as to be expected for the two conformers differing in the sense of the tilt angle of the two phenyl substituents. Depending on the number and species of substituents, the number of different isomeric conformers increases as does the number of intense peaks. Thus, the entire fine structure is interpreted as overlay of the triple peak features of the various configurational conformers. Obviously, this triple peak feature represents the basic signature of microsolvation of Porphyrin derivatives in helium droplets. Severe line broadening can be induced by strong saturation as obtained by the high photon flux of pulsed dye lasers. The corresponding spectra are added as gray lines in Figure 3. Similar as Phthalocyanine, Porphyrin exhibits exceptionally sharp electronic and vibronic transitions which is ideal for resolving the helium induced fine structure. For both species the vibrational fine structure of the electronic excitation of substituted compounds does not show the characteristic low energy torsional or bending modes of the substituents. The close similarity of the vibrational fine structure of the fluorescence excitation spectrum and the dispersed emission spectra reveal a negligible change of the electron density distribution upon electronic excitation to S_1_.

**Figure 4:**
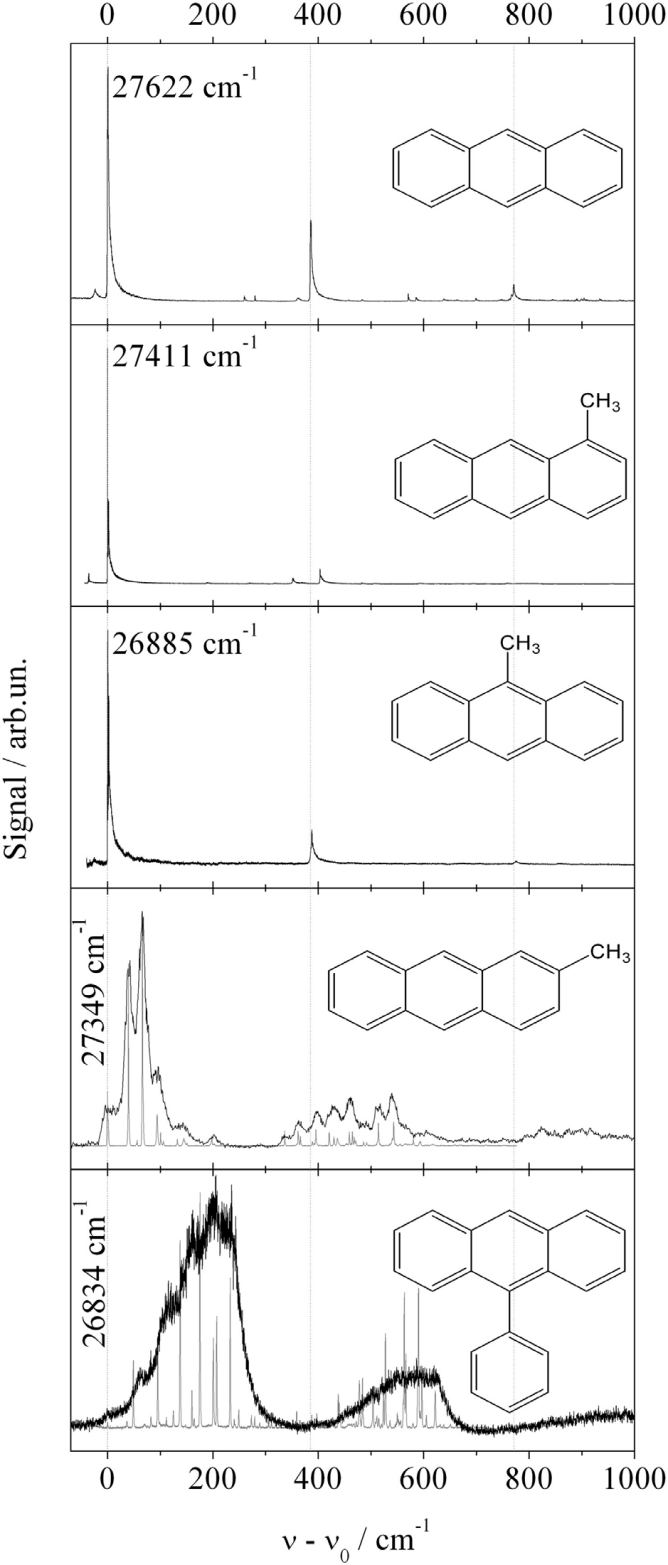
**Fluorescence excitation spectra of fife Anthracene derivatives as indicated in each panel**. Vertical dashed lines indicate basic vibronic transitions present for all derivatives. Torsional mode progressions resolved for 2-MA and 9-PA in the gas phase (gray line) are damped in helium droplets (black line).

The third study investigates Anthracene derivatives. This study includes derivatives where substitution reduces the molecular symmetry. In the case of a single substituent, inversion symmetry is lost and the compound exhibits a permanent dipole moment. For bare Anthracene and the additional four Anthracene derivatives, namely 1-methylanthracene (1MA), 2-methylanthracene (2MA), 9-methylanthracene (9MA), and 9-phenylanthracene (9PA), the fluorescence excitation spectra are shown in Figure 4 (Pentlehner et al., 2011). Roughly, the vibrational mode pattern of bare Anthracene is reproduced for all four derivatives as indicated by the vertical dashed lines. Two of the derivatives do not exhibit low energy progressions (1MA and 9MA) while the other two do (2MA and 9PA). As revealed by the presence of low energy progressions, only the latter two derivatives change the equilibrium configuration upon electronic excitation. For both species, the line widths of the low energy progressions are significantly broadened (black lines) compared to the gas phase spectra (gray lines). In contrast to the Pyrromethene derivatives, line broadening is present throughout the spectrum including the electronic origin. Thus, the damping of low energy modes cannot justify the line broadening. The change of the equilibrium configuration as expressed by the low energy progressions is induced by the electronic excitation and, thus, caused by the change of the electron density distribution. Most likely this change acts not only on the intramolecular nuclear arrangement but also on the arrangement of the helium environment. The latter perturbation may be the reason for line broadening. According to this mechanism, the change of the electron density distribution is the driving force for intra- and intermolecular rearrangements which become effective on the line widths in the electronic spectra of these two Anthracene derivatives. Further details of these spectra are discussed in Pentlehner et al. (2010, 2011); Pentlehner and Slenczka (2012, 2013). Thus, the systematic investigation of Anthracene derivatives provides evidence for the change of the electron density distribution being responsible for helium induced spectral features.

### 3.3. Photochemistry inside superfluid helium droplets

**Figure 5:**
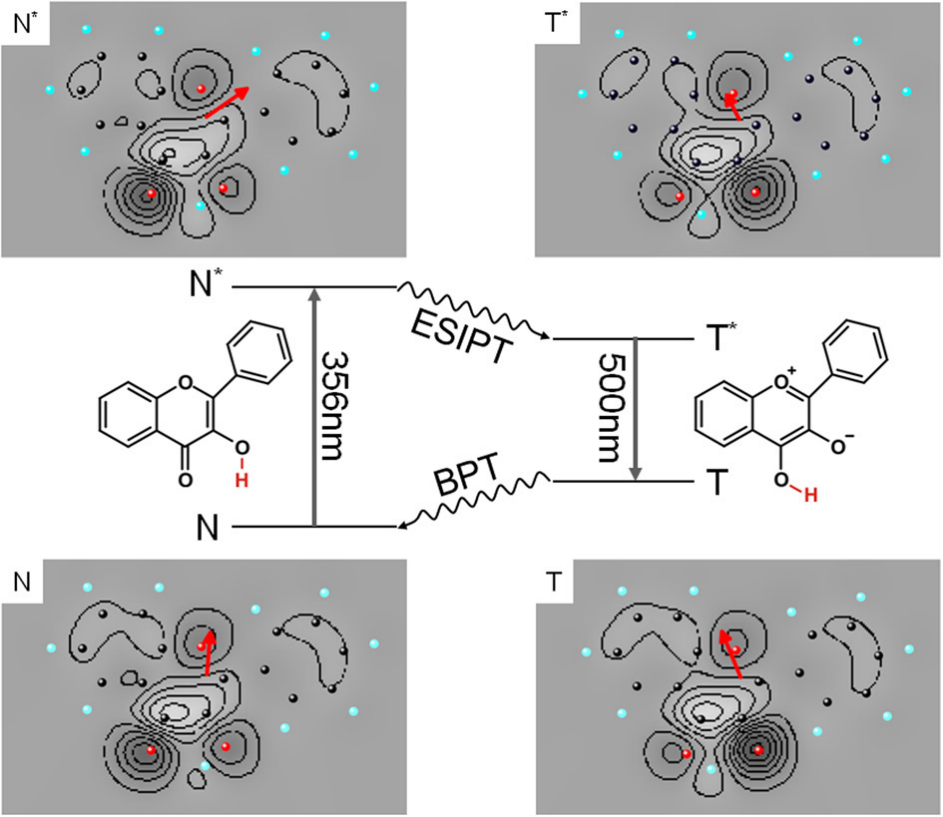
Photocycle of 3-Hf starting with electronic excitation of the normal form (N to N^*^) followed by ESIPT, continued by radiative decay (T^*^ to T) and finished by BPT. The charge density of the four configurations of 3-Hf are depicted as contour plots and the corresponding electric dipole moment is added as red arrow which shows value (length) and orientation (direction).

Our first approach to photochemistry in superfluid helium droplets was the study of the well known excited state intramolecular proton transfer (ESIPT) of 3-hydroxyflavone (3-Hf) and its counterpart in the electronic ground state called back proton transfer (BPT) (Sengupta et al., 1979). As depicted in the center panel of Figure 5, ESIPT and BPT are induced by electronic transition and, thus, by the change of the electron density distribution in accordance with Born-Oppenheimer approximation. As demonstrated in Ernsting and Dick (1989); Muehlpfordt et al. (1994); Ito et al. (1992) the homogeneous line width at the electronic origin of the corresponding fluorescence excitation spectrum reveals the rate constant of ESIPT given that other non-radiative decay paths of N^*^ can be neglected. The homogeneous line width of the corresponding transition in the dispersed emission spectrum is given by the rate constant for BPT and the rate constant for the radiative decay of T^*^. The latter can be determined experimentally from the readily observable radiative decay time. Since in the gas phase a hot tautomer is generated, congestion of transitions of numerous quantum states of the tautomer prevents resolution of the homogeneous line width of individual transitions in the dispersed emission spectrum (Ito et al., 1992). This problem can be overcome by using helium droplets as a host system. The experiment may profit from the highly efficient dissipation of vibrational energy into the helium droplet. Thus, the cooling rate of the nuclear degrees of freedom of the excited dopant molecule which exceeds the radiative decay rate allows to record dispersed emission of a cold tautomer (T^*^). In fact, dispersed emission spectra of the tautomer showed vibrational fine structures, however, only Voigt-profiles with line widths of about 60 cm^−1^ could be resolved (Lehnig et al., 2009; Pentlehner et al., 2011). Even more surprising, the electronic origin and the vibrational fine structure in the fluorescence excitation spectrum nicely resolved in the supersonic jet experiment (Ernsting and Dick, 1989) were entirely washed out in helium droplets (Lehnig et al., 2009). Obviously, in this case the electronic degree of freedom is responsible for the strong perturbation by the helium environment. ESIPT as well as BPT are initiated by purely electronic transitions and, thus, by the change of the electron density distribution. The electron density distributions of the four conformers are shown as contour plots in Figure 5. The corresponding dipole moment is emphasized by the red arrows, indicating its value and direction. Compared to bending or tilting of a methyl or phenyl substituent, proton transfer requires even stronger forces. It is inconceivable that changes of the molecular polarity as induced by electronic transitions of 3-Hf should proceed without severe perturbation of the helium environment. As in the case of 2MA and 9PA, it appears to be the change of the electron density distribution which perturbs the helium environment and, thus, induces severe line broadening in the electronic spectra.

The possibility to design molecular complexes with well defined stoichiometry and the option to distinguish even isomeric variants of such complexes allows one to study the influence of solvents on photophysical processes on a molecular level. Since the influence of polar or protic solvents on the ESIPT of 3-Hf is well known (Sengupta et al., 1979; Ito et al., 1992), we have investigated 3-Hf-(H_2_O)_*n*_ clusters in helium droplets (Lehnig et al., 2009). In a gas phase experiment it was shown that a single water molecule suffices to suppress ESIPT entirely (Ito et al., 1992). More recent gas phase experiments come to the conclusion that at least two H_2_O molecules are needed to block ESIPT (Bartl et al., 2008, 2009). In contrast, the helium experiment unequivocally reveals that one or two water molecules do not affect the 100% efficiency of ESIPT. This was revealed by dispersed emission spectra showing exclusively the signal of the tautomer (Lehnig et al., 2009). Only for an average amount of 4 or 5 water molecules a signal contribution of the normal form N^*^ of 3-Hf could be recorded in helium droplets. All may depend on the configuration of the 3-Hf-(H_2_O)_*n*_ clusters present in the various experiments. According to our calculations which were performed without the helium environment (which means under gas phase conditions) only one stable configuration of a 3-Hf-H_2_O complex was found. For this complex the water molecule is merged into the proton transfer coordinate. For this complex concerted proton transfer proceeds under similar energetic conditions as for bare 3-Hf (Pentlehner et al., 2011). For the complex with two water molecules one can imagine the same 3-Hf-H_2_O configuration with one additional water molecule attached or a chain of two water molecules inserted into the proton transfer coordinate. According to our calculations both configurations allow for concerted proton transfer under energetic conditions similar to bare 3-Hf (Pentlehner et al., 2011). Obviously, calculations of ESIPT for the water complexes without the helium environment (which means for gas phase conditions) are in contradiction to the experimental observations under gas phase conditions. However, they are in agreement with the experimental observations in helium droplets. Recent data recorded in helium droplets from deuterated samples of bare 3-Hf and in addition from all possible combinations of deuterated and protonated samples of 3-Hf and water molecules have shown identical ESIPT behavior as for the purely protonated 3-Hf. At this point one may raise the question on the complex configuration in the helium droplet experiment. The missing influence of one or two water molecules on the ESIPT may indicate that the 3-Hf molecule is shielded by the helium solvation layer. Thus, the water molecules are separated by the helium layer and ESIPT remains unaffected. Only for an average amount of 4 or 5 water molecules the shielding by the helium layer is overcome. Alternatively, the helium environment may favor exclusively those configurations which allow for concerted proton transfer for the 3-Hf-water complex with less than fife water molecules. Finally it should be noted that both the tautomeric and the normal emission (the latter observed for clusters with more than four H_2_O molecules) were spectrally very broad. In the case of ESIPT and BPT of 3-Hf and of its clusters with water, the experimental observations of severe line broadening were counterintuitive. Again, the possible mechanism may be the change of the electron density distribution which simultaneously drives the proton transfer and perturbs the helium environment. The latter explains line broadening.

## 4. Discussion

Electronic spectroscopy provides insight into microsolvation in superfluid helium droplets. Detailed information is revealed by the spectral fine structure of the ZPL and of the accompanying PW. The electronic spectrum of Glyoxal reflects what is expected for a molecule when doped into a superfluid helium droplet. The ZPL reveals the rotational fine structure of an asymmetric top rotor while the PW reflects the spectral structure of elementary excitations of superfluid helium (Hartmann et al., 1996a; Pörtner et al., 2002). However, in this respect Glyoxal is exceptional. All other molecules or molecular complexes investigated so far show a ZPL which is either single peaked or exhibits a helium induced fine structure other than free rotation in a quantum fluid. The PW comes up with a spectral shape in the range from very broad and unstructured to rather narrow in the width consisting of a series of peaks sometimes as sharp as the ZPL. Empirically these features are easily justified by the also empirical model of a non-superfluid helium solvation layer covering the surface of the dopant species. Consequently, we deal with a helium solvation complex dissolved into a superfluid helium nanodroplet. Thus, the PW may consist of excitations of the helium solvation layer with possibly rather sharp transitions (known as van der Waals modes) in addition to excitations of the helium droplet body, both coupled to electronic excitation of the dopant species. A helium induced fine structure of the ZPL is explained by the presence of more than only one configuration of the helium solvation complex. Thus, the spectral position and spectral shape which are similar for the fine structure of the ZPL and van der Waals modes are not anymore the discriminating criteria of ZPL against PW. Consequently, other criteria need to be established in order to provide an unequivocal assignment of the helium induced spectral features. As shown also for Glyoxal (Hartmann et al., 1996a) in many cases the oscillator strength of the ZPL exceeds that of the PW which becomes effective in a different saturation behavior of both signals. And in contrast to the ZPL at the electronic origin, the PW exhibits only red shifted emission because of the dissipation of the phonon energy prior to radiative decay. Vice versa, a coincidence of the origin in the dispersed emission spectrum with the excitation frequency is an unequivocal criterion for the ZPL. This criterion confirmed the presence of two different species responsible for the doublet splitting in the ZPL of Tc (Pentlehner and Slenczka, 2012) and also to identify the number of isomeric configurations of Pc-Ar clusters designed in helium droplets (Lehnig et al., 2007). If ZPL and PW are merged into a single helium induced fine structure (as shown for example in Figure 3 of this manuscript) the problem in the assignment of ZPL and PW is in the first place the missing of the phonon gap which separates the PW of superfluid helium from the preceding ZPL. Secondly, electronic excitation accompanied by a significant change of the shape of the dopant species may lead to an oscillator strength of the PW dominating over the ZPL as reported in Loginov et al. (2005). The change in the shape of the dopant species can either be a nuclear rearrangement or a change in the electron density distribution or both. Thirdly, transitions of metastable configurational variants of a helium solvation complex do not necessarily exhibit oscillator strengths which all exceed that of the PW. Finally, the electronic excitation of such complexes may further reduce the configurational stability. Thus, even without the presence of excess excitation energy the excited complex may undergo relaxation prior to radiative decay. In this case, even a ZPL may show red shifted emission. In summary, the ZPL may show spectroscopic features such as high saturation threshold and red shifted emission which are usually taken as evidence for a PW. Vice versa, the PW may come up with rather sharp spectral features similar as the ZPL which are assigned to van der Waals modes of the helium solvation complex. Thus, experimental criteria to distinguish the PW and ZPL in electronic spectra of molecules in helium droplets do not allow to discriminate van der Waals modes as part of the PW against a ZPL of a metastable solvation complex.

As demonstrated for the Porphyrin derivatives in Figure 3, saturation broadening may hide the helium induced fine structure entirely. While saturation broadening is a technical problem which can be avoided, line broadening induced by the dopant to helium interaction is an intrinsic problem for the application of helium droplet spectroscopy. Established as HENDI spectroscopy (Callegari et al., 2001) with many expectations, the limiting factors need to be discussed and, thereby, might even be turned into a prospect. This will be emphasized in the following discussion by some example spectra. The electronic origin of bare Porphyrin is a prototype for the problem caused not only by saturation broadening but in addition for the problem to distinguish ZPL and PW. The oscillator strength revealed by the saturation behavior and the spectral position were the criteria supporting the assignment of the ZPL and PW (Hartmann et al., 2002). However, the experimental observations taken as evidence for an assignment of the PW do not exclude an alternative assignment to ZPLs of configurational variants of a solvation complex. Similar ambiguities need to be considered for the signals assigned to the PW of Mg-Pc (Lehnig et al., 2004) or Pc (Lehnig et al., 2007). The problem of saturation broadening is nicely exemplified at the ZPL of Porphyrin which consists of a fully resolvable triple peak feature when recorded under appropriate experimental conditions (cf. Figure 3 top panel). The same ZPL has previously been identified as singly peaked already under moderate saturation conditions (Lindinger et al., 2001). The problem of saturation broadening is nicely demonstrated for the entire series of Porphyrin derivatives. It need to be mentioned that in addition to pure saturation broadening the growing intensity of the PW may finally hide the ZPL entirely.

A remarkable example in this context is the electronic origin of TPC shown in the bottom panel of Figure 3. Within the first 10 cm^−1^ the signal can be separated into three parts. The first part is the signal within the first 1 cm^−1^ showing what was identified as the triple peak feature characteristic for the ZPL of Porphyrins in helium droplets (Riechers et al., 2013). The leading intense peak exhibits a line width of only 0.05 cm^−1^. The second part beyond 1 cm^−1^ consists of a series of similarly sharp peaks (cf. black line in the bottom panel of Figure 3) which all exhibit a reduced oscillator strength compared to the ZPL. The third contribution exhibits the smallest oscillator strength and, therefore, can only be recorded upon severe saturation of the first two parts. The gray spectrum in the bottom panel of Figure 3 recorded for high photon flux shows the third part overlapping with the second part and preceded by the first part the latter two with severe saturation broadening. The third part fulfills the characteristic criteria of a PW of the helium droplet body in terms of low oscillator strength, frequency gap to the ZPL, and spectrally broad shape. As discussed above the analysis of the second signal part can not discriminate an assignment to ZPLs of configurational variants of the helium solvation complex against van der Waals modes of the non-superfluid solvation layer. The saturated spectrum plotted as gray line in the bottom panel of Figure 3 shows the ZPL still spectrally separated from the other two - now - congested signal parts. Upon further increased photon flux all three merge into a single peak about 10 cm^−1^ in width. Such a spectrum is shown in Figure 13 of Callegari and Ernst (2011). Besides the problem of identifying the correct dopant species, the interpretation of this spectrum modified by severe saturation broadening leads to conclusions on the properties of the dopant species which are clearly refuted by high resolution spectroscopy.

**Figure 6:**
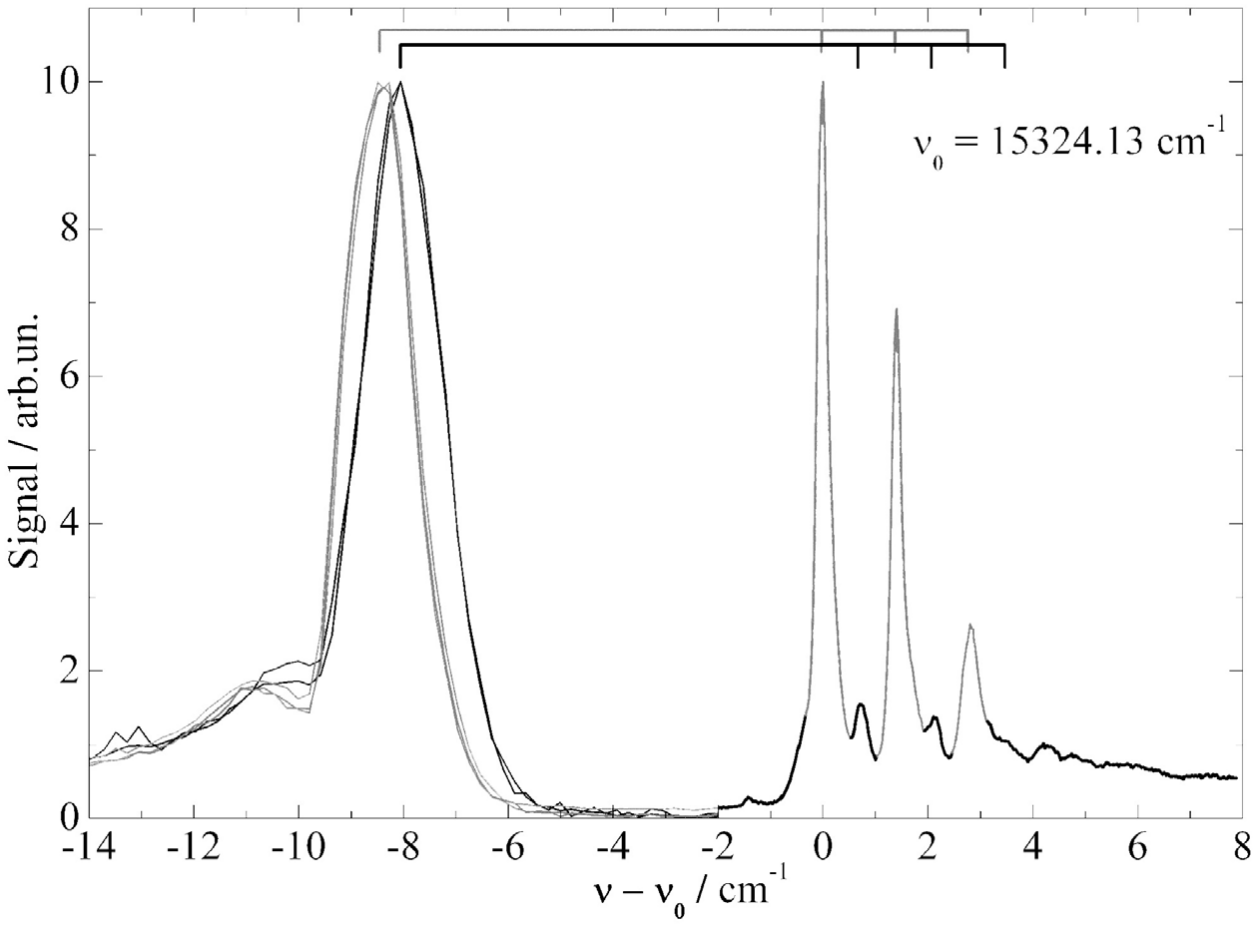
**Fluorescence excitation spectrum (−2 to 8 cm^−1^) and dispersed emission spectra (−14 to −2 cm^−1^) of AlClPc in helium droplets**. Correlation of excitation and emission spectrum is indicated by the two combs.

**Figure 7:**
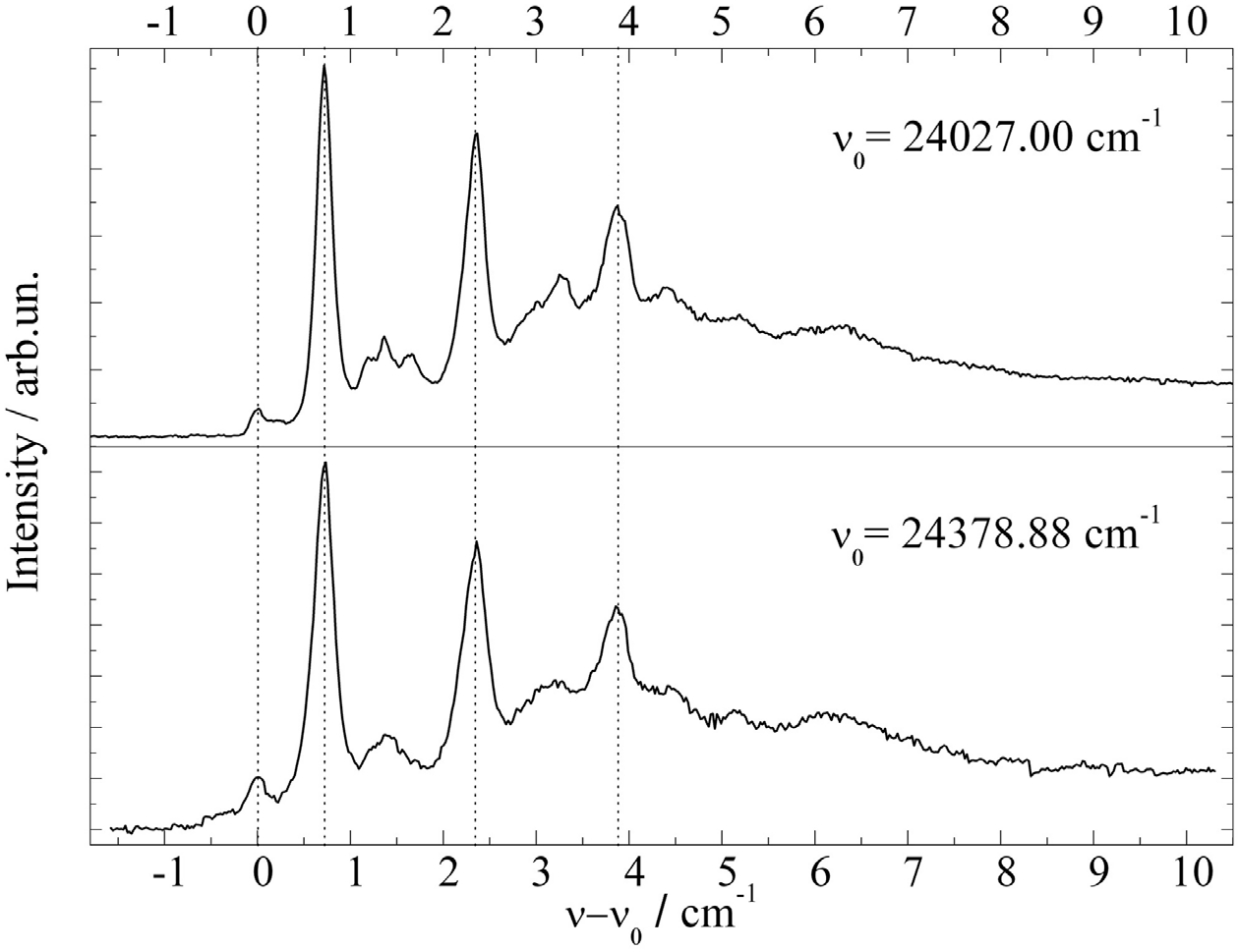
**Fluorescence excitation spectrum of Perylene at the electronic origin (top panel) and at a vibronic transition (bottom panel)**.

In this context two additional examples need to be discussed which are found in the literature (Carcabal et al., 2004; Pei et al., 2007). Both underline the problem of ambiguity in the assignment of PW and ZPL and the problem of saturation broadening. It concerns Aluminum-Chloro-Phthalocyanine (AlCl-Pc) (Pei et al., 2007) and Perylene (Carcabal et al., 2004), whose electronic origins measured in our laboratory are shown in Figures 6 and 7 respectively. In Figure 6 dispersed emission is added in the spectral range below −2 cm^−1^ while for Perylene a vibronic transition is added in the lower panel of Figure 7. Despite the different dopant species, both spectra are dominated by a surprisingly similar triple peak series. However, as the two dopant species are different, the analysis of the two fine structures reveals also very different results. By the help of dispersed emission spectra, the AlCl-Pc spectrum was found to represent two different solvation complexes as indicated by the gray and black combs. Both complexes show almost identical fine structure in the excitation dominated by a series of three peaks. The different intensity of the two signals may reflect the difference in the abundance of the two solvation complexes. The frequency shift of both systems of about 0.7 cm^−1^ is also reflected by the corresponding dispersed emission spectra as indicated by the combs in Figure 6. The red shift of the emission of 8.5 cm^−1^ reveals the relaxation of the solvation complex configuration prior to radiative decay. AlCl-Pc is an example for red shifted emission even upon excitation at the ZPL at the electronic origin. When measured with the high peak power of a pulsed dye laser (certainly not for the purpose of resolving the helium induced spectral signature) much of the fine structure remains hidden (cf. Pei et al., 2007). In contrast to AlCl-Pc, the entire fine structure resolved for Perylene exhibits only one common emission spectrum as shown in Lehnig and Slenczka (2005). The origin of the emission coincides with the first tiny peak shown at the origin of the wavenumber scale in the upper panel of Figure 7. When recorded with increased photon flux, all the tiny resonances in between the dominant trio as well as the leading tiny peak are missing. Consequently, the real origin is missing which causes a false assignment of the electronic origin (cf. Carcabal et al., 2004). Despite all the additional information gained from high resolution excitation spectra and dispersed emission spectra an assignment to either a series of ZPL of variants of a solvation complex or to van der Waals modes of the solvation complex remains open for the fine structure of both molecular dopant species.

The issue of configurational variants as discussed for a single dopant surrounded by a helium solvation layer includes van der Waals complexes inside superfluid helium droplets. It addresses in particular small complexes consisting of a single chromophore and less than 10 additional particles such as rare gas atoms (other than He) or small molecules as published for Tc-X_*n*_ (X; rare gas, H_2_O, and D_2_O) (Hartmann et al., 1998; Lindinger et al., 2006) or Pc-Ar_*n*_ complexes (Lehnig et al., 2007). For electronic excitation the relaxation of a metastable configuration prior to radiative decay and the observation of van der Waals modes need to be considered. Consequently, we are facing the same ambiguity in the assignment of ZPL and PW. Moreover, the presence of a helium solvation layer may support cluster configurations which are entirely absent in the gas phase. Besides the promotion of metastable sites by the helium environment and the low temperature, we need to consider a complex configuration where the noble gas atoms or small molecules reside on top of the helium solvation layer instead of being directly attached to the chromophore. Cluster signals with negligible spectral shift with respect to the bare chromophore and drastically reduced dissociation energies as compared to the gas phase provide evidence for such complexes (Lehnig et al., 2007). In the ultimate case multiple particle doping may thus produce numerous individual particles inside one helium droplet shielded from each other by a helium solvation layer. In contrast to the formation of a large cluster inside the helium droplet this phenomenon is addressed as foam (Przystawik et al., 2008; Goede et al., 2013).

While line broadening as a result of saturated transitions is an avoidable problem, line broadening caused by the dopant to helium interaction is a limiting factor for spectroscopic experiments in superfluid helium droplet and in particular for electronic spectroscopy. As was known from the very beginning, low energy and large amplitude vibrational modes are usually efficiently damped by the helium environment (Hartmann, 1997). As shown by the series of Pyrromethene dye molecules such a damping may become a limiting factor compared to gas phase studies at much higher temperatures. However, this mechanism does not affect the electronic origin which may show up with better spectral resolution and more details as in the gas phase (cf. Figure 2). In addition to this type of vibrational modes the influence of electronic degrees of freedom constitutes a limiting factor. As revealed by the series of Anthracene derivatives the change of the electron density distribution constitutes a severe perturbation of the surrounding helium which ultimately causes line broadening. The entire field of intramolecular photochemical processes induced by electronic excitation is driven by significant changes of the electron density distribution. As exemplified by the ESIPT and BPT of 3-Hf, the accompanying perturbation of the helium environment prevents resolution of any fine structure within the electronic transition. According to our ongoing investigations of isomerization reactions this problem appears to be a real limitation. The influence of the change of the electron density distribution brings us back to Pc the first example discussed in the previous section. The doubling observed in the dispersed emission of Pc is a remarkable spectral signature and a quantifiable response to the change of the electron density distribution of Pc upon the S_0_–S_1_ transition. In contrast to the total disapperance of any fine structure, such spectroscopic signatures show the power of molecular spectroscopy in helium droplets to study the electron density distribution of molecules and its change upon excitation quantitatively.

Finally, recent experiments on free rotation inside superfluid helium droplets in the time domain revealed surprising results. While innumerable experiments provide beautiful rotationally resolved IR spectra of molecules in helium droplets, the observation of rotational recurrences of a coherent superposition of molecular rotor states as induced by non-adiabatic alignment revealed the absence of any coherence (Pentlehner et al., 2013a,b). These experiments are continued in Aarhus and will provide additional information on the dopant to helium interaction which determines the quantitative understanding of microsolvation in superfluid helium droplets.

## 5. Conclusions

Superfluid helium droplets serving as cryogenic matrix revolutionized high resolution matrix isolation spectroscopy. IR spectra in helium droplets revealed unique properties such as free rotation of the dopant, an ambient temperature of only 0.37 K and the possibility to design cold clusters with well defined stoichiometry (Choi et al., 2006). Moreover, helium droplets immediately found a broad reception for the investigation of elementary chemical processes (Slenczka and Toennies, 2008). Besides a triumphal procession into many fields covering physical chemistry and chemical physics, spectroscopy of molecules doped into superfluid helium droplets provides insight into an exceptional weak dopant to helium interaction and into the phenomenon of superfluidity on an atomic scale. Despite the weakness of the dopant to helium interaction, electronic spectroscopy of molecules in helium droplets reveals very pronounced features in particular in electronic spectra. Of particular interest for the study of microsolvation are the fine structure imprinted into the ZPL and the PW. Sometimes these structures suffer from line broadening. While saturation broadening can easily be avoided line broadening due to damping of low energy and large amplitude motions is an intrinsic problem of matrix isolation spectroscopy. According to the variety of experimental results on electronic spectroscopy in helium droplets, the perturbation caused by the change of the electron density distribution is an additional factor for severe line broadening. Since the change of the electron density distribution is the driving force of many photochemical processes, its perturbative action on the helium environment is a limiting factor for the application of superfluid helium droplets as a host system. What turns out as a limiting factor may change into a prospect for direct observation of the change of electron density distribution accompanying electronic excitation. This is nicely exemplified for the example of Phthalocyanine with an electronic excitation almost imperceptive for the nuclear arrangement but with a very pronounced spectral response induced by the helium environment. Therefore it is of vital interest to further explore the very special dopant to helium interaction. The perturbation induced by the change of the electron density distribution as proposed and discussed for several experimental results may lead us beyond Born-Oppenheimer approximation. In addition, one needs to keep in mind that the assignment of ZPL and PW can not be done beyond any doubt as discussed above. Of particular interest is the spectral signature that can be expected for van der Waals modes of the helium solvation layer. Gas phase spectra of selected van der Waals clusters should provide information on the spectral signature to be expected for such van der Waals modes. Important information on microsolvation of molecules in superfluid helium droplets can be expected from a more systematic investigation of the PW also under variation of the droplet size. Such studies will benefit from dopant molecules which do not exhibit configurational variants of the solvation complex. Much experimental work lies ahead and the most critical reception of empirical interpretations deduced from the experiment are advised for theoretical endeavors.

## Funding

Financial support has been provided by the Deutsche Forschungsgemeinschaft (DFG).

### Conflict of interest statement

The authors declare that the research was conducted in the absence of any commercial or financial relationships that could be construed as a potential conflict of interest.
